# The Value of Expert Testimony in Medicolegal Cases—From the Legal Standpoint1Presented at the thirtieth annual meeting of the Medical Association of Central New York, at Buffalo. October 19, 1897.

**Published:** 1898-03

**Authors:** Tracy C. Becker

**Affiliations:** Buffalo, N. Y.


### BUFFALO MEDICAL JOURNAL.

VOL. XXXVII. MARCH, 1898. No. 8.

### Original Communications.

### THE VALUE OF EXPERT TESTIMONY IN MEDICO-LEGAL CASES—FROM THE LEGAL STANDPOINT.[1]

By TRACY C. BECKER, Esq., Buffalo, N. Y.

I ASSUME that I am to discuss the value of expert testimony in courts of justice and the first question is, what do we mean by expert testimony? It is, in brief, opinion evidence; a statement of matters of opinion as distinguished from matters of fact. There are many kinds of expert testimony besides the testimony of expert medical men. The border line between the statement of a fact and the statement of an opinion, based upon facts as the result of observation, is a wavering one. As I have pointed out in another place (Witthaus and Becker's Medical Jurisprudence, etc., Vol. I., Chap. V., p. 50,) there are many statements made by witnesses upon the stand which are matters of opinion, although we call them and treat them as matters of fact; such as, for instance, the statement that a man was intoxicated; or, quite as important, quite as familiar an example, matters of identity. Lay witnesses testify constantly to meeting a person and identifying him. All questions of proving an alibi rest upon that class of evidence, which is really nothing but opinion evidence. And so, without enumerating all the occasions on which testimony is given which involves the expression of opinions, we must assume at the start, I think, that you cannot frame any law, nor can you frame any definition concerning expert testimony, based solely on the declaration that it is merely opinion evidence. All kinds of expert testimony are essential to the administration of justice, because our final tribunal on questions of fact, the jury, is made up of men taken from the body of society. They are men of ordinary education and

1. Presented at the thirtieth annual meeting of the Medical Association of Central New York, at Buffalo, October 19, 1897.

experience and are always, and unavoidably so, without any special scientific training or knowledge concerning the matters which they are to determine and concerning which expert testimony, in the common acceptation of the term, is permitted. This is the reason why expert testimony is allowed, and it is necessary or it never would have been allowed, for it is an exception to the general rule that in courts of law only such evidence is admissible as directly tends to prove or disprove the existence of a fact under consideration. One man's opinion is, generally speaking, as good as another's, unless he has had more extensive training and acquired superior skill and knowledge which enable him to form a more accurate judgment and a sounder judgment. In short, the value of an expert's opinion depends upon three factors: first, what is his own value, meaning his ability and his honesty; second, what are the established facts in the case upon which he bases his opinion; and third, what have been his opportunities for examination of those facts?

This brings us to the consideration of the question, what value have expert witnesses? And if not so great a value as they ought to have, why is it so and what is the remedy?

In discussing this question and presenting the information which I have obtained upon the subject from the highest experts in our profession—namely, the judges of our courts, particularly of our supreme court, I have assumed, and these judges have in the main assumed, that by an expert witness we mean a medical witness, and that upon general questions of science and skill and the like, it is not possible at present to formulate any just regulation of his status. Many letters from the judges were written to me in confidence. Those which have been so written I shall be obliged to withhold, but others have not been written under any seal of confidence. They have been written with knowledge that they would be used on this occasion, and I take the liberty of reading some excerpts from them, not in full, as your time will not permit it, but I presume that if it is within the rules of your society they will be published in full at the proper time and in the proper place.

As it is customary for the junior judges in delivering opinions to be called upon first, I will read a brief letter from Judge Cohen, just appointed to the supreme court bench in the city of New York. Judge William N. Cohen, who in his profession stood very high as an able, distinguished and conservative lawyer, writes:

NEW YORK, October 17, 1897.

*Tracy C. Becker, Esq., Buffalo, N. Y.:*

DEAR SIR—I have rather decided views on the subject referred to, believing it to be the rule that such testimony is quite valueless and that it is essential to eliminate that such witnesses are *retained* in order that proper and just weight may be given to it.

Very truly yours, WM. N. COHEN.

The venerable Judge McAdam, of New York City, writes :

NEW YORK, October 16, 1897.

*Tracy C. Becker, Esq.:*

DEAR SIR—So much has been written on the subject of expert testimony in medico-legal cases that it is impossible for me to add anything of value. If experts in such matters could be induced to agree, their testimony would be invaluable, because settling the question at hand ; but when they disagree, and they often do, its value is so much impaired that lay minds generally apply their own experience and determine the question according to their own judgment, so that in the end the laity prevail and miscarriage of justice is often the result. This is particularly so in insanity cases. Would it were otherwise.

Yours truly, DAVID McADAM.

Judge Werner, of Rochester, who has given this subject much thought and attention, writes as follows :

ROCHESTER, N. Y., October 18, 1897.

*Hon. Tracy C. Becker, Buffalo, N. Y.:*

DEAR SIR—Since writing you the other day I have given the subject of remedial legislation, in regard to expert testimony, considerable thought, but have arrived at no satisfactory conclusion. The evils attending the present method of eliciting medical expert testimony are obvious, but the remedies for these evils are not so apparent. We no doubt all agree upon three fundamental propositions : first, that medical expert testimony is practically indispensable in certain cases ; second, that the present methods of eliciting such testimony are entirely unsatisfactory ; third, that the proper and expeditious administration of justice, as well as the good name of the medical profession, require some reform in these methods.

The evils of the present system which obtrude themselves upon the notice of the two professions and of the public are, first, that experts who are employed upon one side of a case or upon the other, become sometimes unconsciously, and more often deliberately, partisans who are as interested and zealous as the lawyers themselves in behalf of the theories for which they contend ; second, the efforts of counsel on whose side the experts are employed to elicit not the whole truth, but only such facts as will help their side of the case ; third, the tedious and often senseless cross-examination, due in some instances to a lack of previous knowledge on the part of counsel as to theories which are to be advanced by the medical experts, and in others to a lack of preparation and understanding on the part of counsel upon the real medical questions at issue, in either event resulting in great waste of time and usually serving to befog rather than to enlighten courts and juries.

As I have already suggested, it is exceedingly difficult and it may be impossible to provide a satisfactory remedy. It has occurred to me that in criminal cases where the alleged insanity of the defendant, or the employment by him of unlawful methods to effect death or injury, which are the subject of expert medical knowledge, are the questions at issue, the problem might be solved by the enactment of a law providing for the appointment by the court of a commission consisting of not less than three physicians, and in capital cases of not more than five, whose compensation shall be fixed and certified by the court and paid by the county in which the trial is held. The report of the commission, or in case of failure to agree as many different reports as there may be, to be filed a given number of days before the trial in the office of the clerk of the county where the trial is to take place. Upon the trial such reports to be read by one of the persons subscribing the same and each member of the commission to be called for such further explanatory or cross-examination as may be desired. The jury to pass upon such evidence as at present. Counsel on either side to have the right to examine the report or reports after it is filed and before the trial. No physician or expert to be appointed upon a commission by the suggestion of counsel on either side.

I submit this hasty and crude suggestion in the hope that it may point the way to something more satisfactory.

In several cases the problem is immeasurably more complicated. I assume that it will be impracticable to exclude from the witness stand the attending physician or physicians of a plaintiff whose injuries or illness are alleged to be due to the negligence of a defendant. When such physician or physicians are called they are invariably treated as experts. In such case there is no escape from the conclusion that a defendant has the right to rebut their evidence by further expert testimony. The physical examination now provided for by the code is a step in the right direction ; but it is open to the objection that the physicians making the examination are appointed upon the suggestion and nomination of the defendant.

It occurs to me that this matter might be simplified somewhat by providing that in every action for damages, on account of alleged negligence of a defendant, plaintiff shall be required within twenty days after the cause is at issue to make a motion to the court, upon notice to the other side, for leave to take the testimony of the attending physicians before a referee, whose fees shall be at the statutory rate and be paid by the county in which the trial is had. That opposing counsel may be present and cross-examine the witnesses on the hearing before such referee. That the testimony so taken shall be read in open court and all objections to questions passed upon by the court before the answer is read in the presence of the jury. That in addition to this, either party may, at the time when the motion is made for leave to take such testimony, apply for a physical examination of the plaintiff by not more than three physicians, whose report shall be filed with the clerk of the county at least a given number of days before the beginning of the term for which the case is first placed upon the calendar; such report to be open to the inspection of counsel on both sides and the physicians making the same to be called for such further or explanatory evidence as may be desired.

To meet the contingency that in some cases the parties might have more than three attending physicians, it might be advisable to limit the number of attending physicians to be called to three, or if that is impossible, then to increase the number of physicians to be appointed by the court for physical examination to the same number as the attending physicians. I think it will be conceded that even if under some such a plan as this the same number of physicians were called, as under the present system, it would take much less time to adduce their evidence in open court than at present.

In conclusion I must apologise for these very unsatisfactory suggestions. While I have given the subject some thought, the difficulties in the way of improvement were not clearly presented to my mind until I received your letter. The pressure of other duties since then has made it impossible for me to devote as much time to it as I would like. Trusting that this tardy response to your letter may not be too late and that it may be of some slight assistance to you, I remain.

Sincerely yours, W. E. WERNER.

Judge Titus, of Buffalo, who has served many years upon our supreme bench and has had a great deal of experience both in criminal and civil cases, writes as follows :

BUFFALO, N. Y., October 13, 1897.

*Tracy C. Becker, Esq.:*

DEAR SIR—Your favor of the 12th inst., in which you say you are to take part in a discussion before the Medical Association of Central New York in this city, October 18th, on the question of the value of expert testimony in medico-legal cases, and asking my views on the subject, came duly to hand.

It is generally conceded by the profession, that such testimony under the present system of giving it,—each party procuring witnesses favorable to himself and paying for their services,—is not only valueless but absolutely immoral in its tendency. I speak from common observation when I say that such witnesses are influenced largely by their employment and often to such an extent as to become zealous partisans of the party employing them, and in many cases under the guise of expressing an opinion, I believe, commit perjury, and it cannot be corrected too soon.

I know of no way of accomplishing this unless by legislative enactment, which should, in substance, provide that in all cases where medical testimony is to be used parties should be required to make application to the court, on notice to the opposing party, for the appointment of two or more learned men in the medical profession to make an examination of the subject or person and report to the court, with the privilege to either party to call such experts, without any other compensation than such a reasonable sum as shall be fixed by the court, not exceeding a specified allowance per day, say $20, which sum so fixed should be paid by the county.

I have not given sufficient thought to the remedy for the evil which we all recognise to give advice upon this subject and my ideas are somewhat crude, but I believe great good can be accomplished by legislation along the lines I have indicated.

Yours truly, ROBERT C. TITUS.

Judge Goodrich, of Brooklyn, writes as follows :

BROOKLYN, October 14, 1898.

*Tracy C. Becker, Esq. :*

DEAR SIR—Yours of the 12th received. The only suggestion which my limited leisure allows me to suggest was one which I made to a cognate society in New York last winter in an after-dinner speech—that there ought to be public experts appointed by the governor. This to save the ridiculous experience which all of us have seen of paid experts in criminal cases swearing for or against the sanity of a prisoner, according to the amount of their fee and the person who calls them.

Yours truly, W. W. GOODRICH.

Judge Gerrit A. Forbes, who has also been quite a number of years upon the bench and has a large experience in cases involving the giving of expert testimony, writes as follows :

CANASTOTA, N. Y., October 14, 1897.

*Tracy C. Becker, Esq., Buffalo, N. Y. :*

DEAR SIR—I wrote a letter recently to some one (I don't now remember whom) approving to the fullest extent the attempt to secure legislation and the appointment of commissioners to be used by the court in producing expert testimony in medico-legal cases.

I am convinced that such legislation would be of great and valuable service to the bench as well as to the bar; first, for the reason that competent persons would be selected, and secondly, that the commissioners would be nonpartisan. The danger has always been that the expert, when called by a party to testify, in too many cases becomes partisan and for the compensation which he receives tries to make his services valuable to his employer to the exclusion of the other side, and juries have less faith in that class of testimony for that reason.

There are a great many high-minded men who are called as legal experts and to whom the court readily gives credit, but the jury isn't always so certain to give them the credit which they deserve. There are very few instances of the other kind, I am glad to say, and the court seldom has any difficulty in distinguishing the right from the wrong class. Sincerely yours, GERRIT A. FORBES.

Judge Adams, of Canandaigua, a distinguished member of the appellate division of the supreme court, who has had very large experience both as a practitioner at the bar and upon the bench in cases involving the use of expert testimony, writes:

CANANDAIGUA, N. Y., October 18, 1897.

*Hon. Tracy C. Becker, Buffalo, N. Y.:*

DEAR SIR—Your letter of the 12th inst. reached here while I was engaged in the performance of my official duties at Rochester and from which, as you know, I was not relieved until Friday evening. Consequently I have had no opportunity to give any attention to the subject-matter of your inquiry until today.

The unreliability of expert testimony is certainly something which demands careful consideration at the hands of every serious-minded and thoughtful member of the profession. For it must be apparent to even the casual observer that so-called expert witnesses oftentimes fall far short of furnishing evidence which is entitled to the slightest consideration, or which may be regarded as at all helpful to either courts or juries. This condition of affairs, I am inclined to think, arises from the fact that the moment an expert is employed in a given case he identifies himself with his employer and in a very short time, unconsciously perhaps, becomes such an extreme partisan that his testimony amounts to little more than an argument in support of his client's cause. Indeed it often happens, as you very well know, that "expert witnesses" are far more anxious to destroy each other than they are to elucidate the particular question to which their attention has been directed. And therefore it is, as was said by a distinguished member of the bench in an elaborate opinion quite recently written, that

If it should appear that his (the expert's) opinion is formed without the aid of facts necessary to enable him to come to a conclusion, that opinion may be disregarded however confidently it is testified to by the witness. (Justice Rumsey in Weber vs. 3d Ave. R. R. Co., 12 App. Div., 512–515.)

But it is unnecessary to dilate further upon the unreliability of expert testimony, which every lawyer of experience recognises and deplores ; the real question being, what remedy can be applied to the evil ?—and as to that I hardly know what to say.

It has been suggested that a certain number of experts should be employed and paid by the state, upon the supposition that this would deprive them of any motive for bias in their opinions. While this proposition possesses some commendable features, it seems to me for obvious reasons wholly impracticable, and therefore I am not prepared at present to advise its adoption ; and, at the same time, I have no other plan to suggest, unless it be for the courts to continue their denunciation of so-called expert testimony until a public sentiment is aroused which shall be sufficiently potent to compel experts to mend their ways and to aid rather than deter the proper administration of justice.

I shall be interested in the outcome of your discussion, and if it results in proposing some legislation on the subject, shall be glad to know what it is.

Regretting that I cannot be of some assistance to you, although I am sure you do not require any, I remain,

Yours very sincerely, WILLIAM H. ADAMS.

Judge Rumsey, whose opinion as a member of the appellate division in the first department was just quoted by Judge Adams, who stands high as a member of the bench of this state and also as a text-writer, writes as follows :

BATH, N. Y., October 16, 1897.

*Hon. Tracy C. Becker :*

DEAR SIR—I received your letter of October 12th and I have been somewhat at a loss just how to answer it. I have a pretty decided opinion about the value, or rather the worthlessness, in most cases of so-called expert evidence. I do not mean by this to say that I discredit or undervalue the sworn opinion of an unbiased witness, not subsidised by either side but simply testifying to his honest judgment upon the facts which he has observed or which are presented to him, but unfortunately such expert testimony is very seldom given. As we all know, an expert witness is a man who has been consulted by the side for whom he is sworn, often retained by them, and who understands that it is a part of his contract to so present the facts, or present only such facts, as will sustain the opinion favorable to his client, and to state that opinion to the jury under his oath ; the sole difference between him and an advocate being that he makes his argument under oath and that he endeavors to add the weight of the oath to the opinion of the advocate.

Such testimony is, in my judgment, not only to be discredited, but it is discreditable. In a very considerable experience as a lawyer and a judge I have seen a good deal of that sort of thing and I am of opinion that it is not only becoming a crying evil and a danger in the administration of justice, but a disgrace. It is almost always the case now, that upon every trial where the opinion of witnesses can be of any service, those who are called upon to express opinions deliberately range themselves upon one side or the other and argue as to the weight to be given to their opinions from the witness stand precisely as counsel argue, standing at the bar. Of course this is all wrong and some means should be taken to remedy it.

More than twenty years ago I drafted a bill which I procured to be offered in the senate, providing for the selection by a judge of certain experts whenever, in any given case, testimony of opinions should be necessary and competent. The substance of it was, that upon motion by either party a judge might select a given number of experts, who should inform themselves in regard to the case and hold themselves in readiness to give testimony ; that these experts must be called by the district attorney ; that they should not be at liberty to consult with either party as to the facts or as to the opinion which they were to give, nor should they be selected upon the nomination of either party. The bill provided further that either party might swear other experts in the usual way, but that those experts should not be entitled to any pay from the people, no matter upon which side they were sworn. The bill, however, failed to go through and I dare say it ought to have done so. I am not prepared to say that any such system is advisable or even practicable. I have thought, however, seriously lately, whether it would not be advisable upon the whole to prohibit by statute the swearing of any subsidised expert witness, but require that if any such person was to be presented to the jury, that he should be at liberty to give his opinion upon the facts which were made to appear by the testimony, with the understanding that he was not swearing to that opinion, but he was making an argument about it, precisely as a lawyer would make an argument upon a question of law, and that his argument, like the argument of counsel, might be rejected by the jury if they saw fit to do so.

I am quite clear that some steps should be taken to limit the present system of examination of experts, and to bring it back to what it used to be when an expert witness was a witness and had not been educated to regard himself as a paid counsel, whose duty it was to win the case if he possibly could do so. If that could not be done, I am rather inclined to think that a better way would be to prove the facts to the jury and allow the expert witness to state his opinion upon the facts and his reason for that opinion, as suggested above, just as any other counsel would ; it being understood that his opinion would have the same weight and no more as the paid for and unsworn opinion of anybody else. Such a rule might be applied without much bother, as it seems to me, and I have no doubt that it eventually would work a considerable and necessary reform. Yours very truly,

WILLIAM RUMSEY.

Judge Edwin A. Nash, of the seventh judicial district, who is considered one of the most able and conservative of our judges, writes briefly as follows :

Avon, N. Y., October 15, 1897.

*My Dear Mr. Becker :*

It has been my experience that in medico-legal cases expert testimony is always a great aid to a full understanding of the case. Whether any legislation would prevent the conflict of opinion which we often have between medical experts on one side and the other is something to which I have not given much thought. As medicine is not an exact science it would seem to be impossible to procure a body or committee of physicians who would agree in a case not the subject of demonstration or exact diagnosis.

It seems to me that courts and juries must always be left to exercise their judgment as to the character and standing of the experts called as witnesses on either side, the knowledge they exhibit of their profession and the case, and the degree of candor and fairness with which they give their testimony.

These views will readily occur to you as those which will be entertained on one side of the question as to whether any legislation upon the subject would be at all practicable.

Very truly yours, EDWIN A. NASH.

The venerable Judge Daly of New York, whom we all admire so much, writes as follows :

New York, October 15, 1897.

*My Dear Mr. Becker :*

I have only time for a hurried reply to your valued letter of the 12th, so that you may get this before the date fixed for your lecture. In respect of the subject named, I can only say that the difficulties in the way of carrying out any of the schemes of remedial legislation which I have heard or read about, so as not to work injustice, seem to me so great that I am inclined to prefer that the law and practice with regard to medical expert testimony and evidence should be left where it is. There does not seem to be any greater reason for changing it than there is for altering the law with respect to the testimony of experts in the law, in chemistry, engineering, or any other science. Do they not at present stand on about the same footing ?

Yours always, J. F. DALY.

Having read from the expert opinions of our judges, I take the liberty to present a letter from my colleague, Professor Rudolph A. Witthaus, generally estimated by courts and lawyers and by his own profession to be among the most eminent of experts, in the toxological line at least, in this country. He has certainly had vast experience and has been quite as frequently engaged by the state as by the defense in criminal cases. He writes :

NEW YORK, October 14, 1897.

*Dear Mr. Becker :*

I have yours of 10th inst. asking for an expression of opinion upon the present condition of expert testimony and for remedial suggestions. That our present system is bad, very bad, is patent. It could hardly be made worse. But, unfortunately, it is not, in my opinion, possible to find a complete remedy which is practicable.

Three causes contribute to the just disrepute of expert testimony : (1) The fact that the value of such evidence as is submitted is determined by a jury of persons entirely ignorant of the matters discussed, who are instructed—if they be at all—upon such matters by the experts themselves, and who naturally either determine in favor of that side which produces the greater weight of heavy ordinance, or throw the expert evidence out of consideration entirely. (2) The employment frequently of pushing, blatant, ignorant persons (or even of persons who do not hesitate at plain perjury) as alleged experts, not only by defendants, but even by the state in capital cases. (3) The fact that experts are employed, not by the court, but by the parties in interest or by the prosecuting officers, and that, being so employed, they, although sworn to tell the whole truth, are only permitted to tell such portion thereof as may be of service to the client of the examining counsel. And in this connection I may say that the bar is as much responsible for the existing condition of affairs as the doctors and others. In all my employments I have found the task of keeping the counsel who was to examine me in chief within the bounds of my convictions, much more troublesome and difficult than the most searching cross-examination. The inexperienced or incautious expert has more to fear from the counsel whose client is his own than from the opposing counsel.

I know of but one radical remedy for the present condition of affairs and that I fear can only be reached through an abridgment of the jury privilege, which would, however, operate in the interest of innocent persons in criminal actions and of the rights of those of a civil character. All matters calling for expert evidence should be submitted to a jury or commission of men, themselves experts upon the questions involved, which jury or commission—appointed by the court—should find finally upon such questions. Their finding should be transmitted to the lay jury —the final arbiter upon the entire case—as a fact proven. For instance, in a case of poisoning, a commission of state officers (with representation of the defendant, if you please,) find that the deceased died from the action of arsenic. The certificate of that fact from such a commission may be accepted by the lay jury, as they would accept the statement of an eye-witness that he saw the defendant fire a pistol, and that with much greater safety to the defendant than if they (as they now do) rely upon their own lack of knowledge of the fact as forming the proof of the fact of death by arsenic.

But all this is a suggestion of that which is practically impossible. I have thought of means of diminishing the present evils—by the creation of state experts and in other ways. But these are all open to the same objections, *i. e.*, that the questions are threshed out in court before lay jurors, incompetent to judge of the merits of the questions involved, who are more deeply influenced by the tricky turn of phrase by an adroit lawyer than by a statement of fact which would be absolutely conclusive to a juror competent to judge.

In sum you may improve the tone of expert evidence somewhat by state regulations and by the same means also facilitate the collection of evidence (as by an improvement in the coroner system) during the earlier stages of the investigation of criminal cases, but the expert will only occupy his proper place in medical jurisprudence when his statements are weighed by a jury who can understand his language and know whereof he is speaking. Yours very truly,

R. A. WITTHAUS.

If I may be permitted to add a few words to what has been so well said, I will say this : I do not believe that within constitutional limitations, or within the limitations of justice, it would be practicable for any legislation to be drafted which would exclude from the witness stand witnesses who are witnesses not only for the purpose of giving opinions, but to testify to facts which they observe. In order, in a criminal or a civil case, to make expert testimony of value, it is essential that the expert shall have had an opportunity of examining the subject under consideration. If it be a toxological case, the expert should have had the viscera or the other parts of the body of the deceased in his possession and should have observed them carefully and should have made a chemical analysis of them. If it be of an injury to the person of a dead or live body by a gunshot wound, a stab wound or the like, there are observations of matters of fact which you could not preclude a witness from testifying to if he had the opportunity to make the observations. On the other hand, where you have witnesses who are strictly experts, who are not testifying to both matters of observation and matters of opinion, but are testifying simply to a hypothetical proposition put to them in the language of counsel, involving the known facts of the case or the facts claimed to be established, on one side or the other, and asking for an opinion, there are no constitutional limitations and, in my judgment, no limitations of practicability or of justice which would prevent requiring such witnesses to be taken from a commission or a board created by law, under the sanction of the law and appointed by some proper appointing power. Just how that could be worked out, as the law now stands, and as the practice now stands and custom and usage now prevail, is a very serious question. There is an element of politics that is liable to enter into a proposition of that kind, which might be infinitely dangerous. Where I had at one time hoped that this whole subject, so far as the criminal side is concerned and to a certain extent in negligence cases, might be worked out, was through a reformation of our coroners' system. For that reason, after considerable thought upon the subject and consultation with eminent members of my own profession and of yours, I did all that I could in the constitutional convention to secure an amendment taking the office of coroner out of the constitution, so that the legislature might substitute something else in place of the present antiquated and useless, if I may use the expression, system of coroners' examinations. The constitutional convention did adopt such an amendment and the people ratified it at the polls and at the next session of the legislature, or the second one after that, a bill was presented, which I drafted, with the assistance of Dr. Witthaus and of the committee on legislation of your state medical society, and of the homeopathic state medical society. This bill died in committee. At the next session (1896) another bill was prepared, under the auspices of the same committees and the same gentlemen, and with the very able assistance of Judge D. Cady Herrick, of Albany. I have a copy of that bill before me. I will not read it to you, but will leave it to be printed in your Transactions, because it embodies what, after a long and careful deliberation, with due regard to the existing conditions and also with due regard to what was practicable politically, as we believed, we drafted and presented to the legislature in the way of a bill, which was as follows :

### STATE OF NEW YORK.

No. 906. Int. 752.

IN ASSEMBLY,
February 14, 1896.

Introduced by Mr. ROBBINS—read once and referred to the committee on the judiciary.

### AN ACT

To abolish the office of coroner and to provide for the performance of the duties now performed by coroners.

*The people of the State of New York, represented in Senate and Assembly, do enact as follows :*

SECTION 1. The office of coroner in the several counties of this state shall be, and is hereby abolished when the terms of office of the coroners in office at the time this act shall take effect shall expire.

§ 2. Coroners' juries, post mortem examiners and coroners' physicians shall be, and they are hereby, abolished when this act takes effect.

§ 3. On or after the first Tuesday of November, 1896, the appellate division of the supreme court for the first judicial department shall appoint four medical examiners and four assistant medical examiners, and assign them or any of them to duty in one or more districts in said department, and from time to time may reassign them or any of them from one district to another, temporarily or permanently, as it may deem best for the public service. Said appellate division of said department may also, in its discretion, before or after said day, appoint two other medical examiners for said department, one of whom shall be an expert pathologist, and the other shall be an expert chemist. The appellate division of the supreme court for the second judicial department shall, on or after said day, appoint two medical examiners for said judicial department, and not exceeding twelve assistant medical examiners. The appellate division for the third judicial department shall, on or before said day, appoint one medical examiner for each judicial district in said department, and not exceeding twenty-nine assistant medical examiners. The appellate division of the supreme court for the fourth judicial department shall, on or after said day, appoint one medical examiner for each judicial district in said department, and not exceeding twenty-five assistant medical examiners. The appellate division in each department, except the first, may also, in its discretion, on or after said day appoint two additional medical examiners for each of the said departments, one of whom shall be an expert pathologist, and the other shall be an expert chemist. In making the appointments of assistant examiners, as herein provided for, the appellate division of each department shall designate the county in which each of such assistant examiners shall have his principal office and discharge the duties of his office, and may, from time to time, assign them to duty from one county to another, temporarily or permanently as it may be deemed best for the public service. The salaries or compensation fixed by the appellate division for each of said assistant examiners shall be a county charge upon the county in and for which he is designated to perform his duties, and in which he shall have his principal office, and shall be paid by the county treasurer of said county in equal monthly installments. The medical examiners and assistant medical examiners appointed under this act, and their successors, shall be duly licensed and registered physicians, and their appointment shall be made according to merit and fitness, to be ascertained, so far as practicable, by such examinations as the respective appellate divisions for each department shall prescribe. They shall be appointed and shall hold office for the term of six years, and until their successors are appointed and qualified, as prescribed by this act. In case of the death, resignation or removal of any medical examiner or assistant medical examiner, the appellate division which appointed him may appoint his successor for the full term of six years. Any medical examiner or assistant medical examiner may be removed at any time for cause shown, by the appellate division of the judicial department which appointed him. He shall be furnished with a copy of the charges made against him, and shall have an opportunity to be heard in his defense. Each medical examiner, and each assistant medical examiner, shall, before entering upon his duties, take and file in the office of the clerk of the appellate division of the supreme court of his department, his oath of office, and shall execute and file in said office, a bond with two sureties, to be approved by said appellate division of the supreme court appointing him, or any judge thereof, in the penal sum of $5,000, conditioned upon the faithful performance of his duties. If any medical examiner or assistant medical examiner shall neglect to qualify or to give a bond as herein provided, for the period of thirty days after notice of his appointment, the appellate division appointing him may revoke his appointment and appoint another medical examiner in his place and stead.

§ 4. The respective appellate divisions of the supreme court for each department shall fix the compensation or annual salary, payable in monthly installments, to be paid to the respective medical examiners and assistant medical examiners appointed by said appellate divisions in the manner hereinafter stated, and may, from time to time, refix and change the same as to future but not to past services. Said appellate division in each department may also make provisions for stenographic and other clerical assistance, and for office accommodations, and supplies for the respective medical examiners and assistant medical examiners within such department, and for the actual expenses of the medical and assistant medical examiners. Said appellate division for each department may also direct what county or counties within such department shall pay all or a proportionate share of the expenses of such stenographic and clerical assistance and office accommodations and supplies, and the same shall be paid as county charges by such county or counties.

§ 5. It shall be the duty of the appellate division of the supreme court of the state to make rules and regulations prescribing the powers and duties of medical examiners and assistant medical examiners appointed by it pursuant to the provisions of this act, and to change, alter and amend the same from time to time as necessity may require. The appellate division of each department may make such additional rules in relation to the examiners and assistant examiners in such department, as it may deem proper.

§ 6. It shall be the duty of every peace officer and of every physician, and of all other persons, to report immediately to the medical examiner or the assistant medical examiner of the district wherein the same occurs, every case of sudden or accidental death, or death by violence, or a body found dead, and the facts and circumstances attending the same. The failure of any physician or of any peace officer, or the willful neglect of any other person, to immediately make such report, is hereby declared to be a misdemeanor and punishable accordingly.

§ 7. It shall be the duty of the examiners and assistant medical examiners to examine into the cause of death of all persons who die by suicide, or suddenly, or by accident, or by violence, or may be found dead within the respective districts or counties to which said medical examiners or assistant medical examiners are assigned to duty, and each medical examiner and assistant medical examiner in each judicial department shall have such powers and shall perform such duties as shall from time to time be prescribed by rules and regulations or orders of the appellate division appointing him. Said medical examiners and assistant medical examiners are hereby authorised and empowered to administer oaths, and to take the ante-mortem statements or depositions of persons about to die from other than natural causes, and also to take the depositions of any person or persons concerning the cause of any death, and transmit the same to the district attorney of the county where any such death occurs. Whenever, during or after an examination by him, as herein provided, it shall appear probable to any medical examiner or assistant medical examiner that any person is criminally responsible for causing the death of another, such examiner or assistant examiner shall forthwith apply to a committing magistrate for a warrant for the arrest of such person or persons, and it shall be the duty of such magistrate so applied to, upon the filing with him of a written certificate of such medical examiner or assistant examiner, that upon an examination made by him there is probable cause to believe, and he does believe, that the person or persons named by him are criminally responsible for the death of any person or persons named, or designated as unknown, in such certificate, it shall be the duty of such magistrate, without further proof to issue a warrant for the apprehension and arrest of the person or persons so charged, and to proceed with the examination of the charge so made in the same manner as in other criminal cases. It shall also be the duty of such medical examiner or assistant medical examiner to report the facts in such cases to the district attorney of the county where such crime has been committed, who shall also have power to issue a bench warrant for the apprehension and detention of the person or persons so charged with causing such death, and proceed to investigate the same before any magistrate within the county as aforesaid.

§ 8. Whenever a person has been arrested upon a charge of criminal liability for causing the death of another person and an autopsy or post mortem examination shall be had upon the body of the person whose death has been so caused, said person under arrest shall have the right to be present in person or by counsel or with not more than two licensed physicians, when such autopsy or post mortem examination is made after his arrest; but neither such person nor his counsel nor any physicians so present representing him, shall have the right to take part in such autopsy or post mortem examination, except to view the same.

§ 9. In any criminal action or proceeding or upon any proceeding arising upon a writ of habeas corpus or in relation to lunatics or habitual drunkards, the judge presiding on the hearing thereof may, by a written order, require one or more of the medical examiners or assistant medical examiners of the state to examine any person, body or thing in any way connected with the issues and matters involved in such action or proceeding, and to testify upon any hearing or trial thereof concerning the same. In any such proceedings not of a criminal nature, the court or judge requiring his attendance shall certify the amount of his compensation for such services, and of his necessary expenses therein, and the amount so certified shall be a county charge, and shall be audited and allowed by the board of supervisors of the county wherein such services are rendered and be paid by the county treasurer. It shall be the duty of a medical examiner to attend upon any trial or investigation in any court in the judicial department in which he may be appointed, upon the requisition of the district attorney of any county in such department, or upon the direction of a justice of the supreme court of such department, and when such attendance is required in a county other than that in which he was assigned to duty and in which his principal office is located, his necessary expenses and disbursements in going and returning from such court and while there, when certified to by said district attorney or by a justice of the supreme court, shall be a county charge upon the county where such court is held, and shall be audited and allowed by the board of supervisors thereof, and shall be paid by the county treasurer.

§ 10. On and after the first Tuesday of November, 1896, the duties and powers of coroners in civil actions and proceedings shall, in all the counties of this state, except the county of New York, appertain to and be vested in the county treasurers of such counties, and in the county of New York, in the city chamberlain of the city of New York, and said respective county treasurers and city chamberlain shall, for the performance of said duties and powers of coroners in said actions and proceedings, be entitled to receive the same fees and percentages as are allowed by law for the performance of such duties by a coroner of said respective counties. Said county treasurers or said city chamberlain may deputise any person to perform said duties and exercise said powers in civil actions and proceedings, but shall be responsible for the faithful performance of said duties and the exercise of said powers by the person so deputised.

§ 11. When services are rendered in bringing to land the dead body of a person found in any of the harbors, rivers or waters of this state, the district attorney of the county in which said body is found may allow such reasonable compensation therefor as a medical examiner or assistant medical examiner who examines such body shall certify is fair and reasonable, and in accord with the services rendered; the said charges shall be paid by the county treasurer of such county upon the said certificate of said examiner or assistant examiner approved by the district attorney. This provision shall not be deemed to entitle any person to compensation for services rendered in searching for such dead body.

§ 12. The salaries of the medical examiners shall be paid by the state treasurer upon the warrant of the comptroller out of any funds not otherwise appropriated. The salaries of the assistant examiners shall be paid by the county treasurers of the counties to which they shall respectively be assigned to duty, and the necessary expenses of the assistant examiners other than those hereinbefore provided for shall be audited and allowed upon the certificate of a justice of the supreme court, that the same are just and reasonable, by the board of supervisors of the county in which they are assigned to duty.

§ 13. Nothing herein contained shall be deemed to restrict or otherwise affect the power and authority of the district attorneys of the respective counties to employ and fix the compensation of experts in criminal cases, as now provided by law.

§ 14. All provisions relating to coroners shall be deemed repealed as to each county of this state, when and as soon as the terms of office of the coroners of such county shall expire; and whenever the term of office of any coroner shall expire, or he shall die, or resign or be removed from office, no successor shall be elected or appointed in his place or stead.

§ 15. This act shall take effect on the first Tuesday of November, 1896.

This bill was reported unfavorably by the assembly committee on judiciary.

I wish to say that, in my judgment, two things defeated this bill in the committee on judiciary. One was the fact that we placed the power of appointment of the medical examiners, which we provided to take the place of coroners, in the appellate divisions of the supreme court and would not allow them to be appointed by some politician. Overtures were made to us that the powers that be, on one side or the other, would abandon their objections to this bill if we would simply take the appointing power away from the appellate divisions of the court and give it to the politicians. We said : "The life of this bill depends upon taking it out of politics. We will do nothing of the kind. The bill must stand or fall on that provision." And it fell.

The other objection to it,—gentlemen, I am ashamed to say it,—came from the corporation lawyers, who came to me personally and said that they could not favor this bill because as the law now stood—think of it !—they could go to coroners and squelch investigations of accident cases, where men had been killed by the negligence of corporations, and save their clients from damages, but that if this bill went through they were afraid that there would be too many negligence cases investigated, and there would be less opportunity for them to stop investigations to bring out the facts and to perhaps render their clients liable. For that reason at least two gentlemen connected with the state legislature said to me : "I can't favor that bill and I shall do all I can to defeat it."

So you see some of the obstacles that we are liable to meet with when we try to bring up any measure of reform that touches that side of the case, and it is difficult to conceive of any legislation that will not to some extent touch it in many instances. If you limit the bill simply to criminal charges of manslaughter and the like, it may be that negligence has been the cause of death and is so great that a charge of manslaughter should be formulated upon it. This bill, in substance, provides that there shall be appointed by the appellate division in each judicial department of the state a certain number of medical examiners, and for their examining all the cases that coroners now examine, and having substantially the same powers that coroners now have ; and it provides that the accused, where he is under arrest in case of alleged homicide or injury at his hands for which he is charged as liable criminally, may be present at the post mortem or other examination of a person injured ; and there are various other provisions of a general nature, which, no doubt, if you care to look at it at all, you will examine when you come to see the bill in print. The general effect of it is to abolish coroners' juries and the office of coroner and put in place of them a system of medical examiners, taken from your profession, appointed, not by politicians, but by the courts, as officers of the courts. It does not pretend to limit the right to call experts, but it does provide that in certain cases the court may, of its own motion, summon these trained and skilful men to give their testimony as experts and give the result of their examinations. That is the best remedy that our skill and experience suggest. By "our" I mean the gentlemen whom I have named here : the committee on legislation of your state medical society and of the homeopathic state society and such men as Dr. Macdonald and Dr. Balch, of Albany, Prof. Witthaus, of New York, Dr. Suiter, of Herkimer, many distinguished medical men in New York city, and Judge Herrick and myself.

Another suggestion has been made, not so far-reaching as this, which I approve of very heartily—that is, that in the large cities of the state, at least, it should be possible for the court to appoint a medical commission or board, consisting, we will say, of one expert toxicologist, one expert pathologist and one expert on insanity, and to refer all questions, when an accident happens or an injury occurs or a homicide takes place, by order of the district attorney or of the magistrate before whom the cases come, for examination to this board, giving them the power to get material into their possession for the purpose of examination, to summon witnesses and examine them and then to familiarise themselves with the situation and to render their opinion in writing, very much as Dr. Witthaus suggests in his letter, and to provide that when that opinion is rendered it may be read in evidence by any party at the trial and that either party may call them for cross-examination in regard to it. That would, I think, be a long step in the right direction. It might not be so cumbersome or so difficult to get through the legislature as a change in the coroners' system ; and yet, after all, gentlemen, when you get right down to the finality of this question, in criminal cases at least, you are always liable to have an ignorant coroner, as in a recent case not over a hundred miles from this bailiwick,—and I do not refer to any coroners in the city of Buffalo either,—who may not know how to conduct a post mortem examination, and who may allow men to conduct it who were not competent. You are all the while encountering a very dangerous situation, hence it looks to me as if the radical remedy was the best remedy of all, and that is to change our coroners' system, to turn them into medical examiners, and perhaps have a central board of paid experts of high skill and experience, in all cases appointed by the court—not elected, not appointed by any politicians, but by the court,—and make them the officers of the court, whose report in writing may be open to either side on the trial of all cases, certainly of criminal cases, and perhaps of civil cases, on the order of the court, substantially as provided in our coroners' bill.

